# A Rare Case of Synovial Chondromatosis in the Proximal Interphalangeal Joint of the Right Index Finger

**DOI:** 10.7759/cureus.68072

**Published:** 2024-08-28

**Authors:** Mukesh Phalak, Abhishek Nair, Rajeev Reddy, Shubhankar Chopra, Sushant Kumar

**Affiliations:** 1 Orthopaedics, Dr. D.Y Patil Medical College, Hospital and Research Centre, Dr. D.Y. Patil Vidyapeeth (Deemed to be University), Pune, IND

**Keywords:** cartilaginous metaplasia, cartilaginous nodules, calcifications, benign tumor, synovial chondromatosis

## Abstract

Synovial chondromatosis is a rare condition that is also known as Reichel syndrome. It is a disorder that affects the monoarticular joints. The proliferation and metaplasia of the synovial cartilage are its defining characteristics. Many tiny, intra-articular osseocartilaginous loose bodies are formed as a result of this proliferation. They are fed by the synovial fluid once they break off from the synovial surface and enter the joint cavity, where they develop into calcification and ossification. If left untreated, it usually causes the afflicted joint to malfunction severely. Large joints such as the knee, hip, elbow, and shoulder joints are frequently the sites of nodular proliferation. Smaller joints such as the hand's interphalangeal and metacarpal joints and thumb are among the more unusual locations. Although the disease usually resolves on its own, conservative management options include painkillers, activity modification, and cryotherapy. Surgical options include synovectomy, which is the gold standard procedure and involves removing the loose bodies. The following case study presents a 60-year-old female patient with a rare instance of synovial chondromatosis. She presented to the outpatient department (OPD) due to escalating pain and swelling in the proximal interphalangeal (PIP) joint of her right index finger, which significantly restricted her range of motion. On the hand X-ray, several small, uniformly sized calcified bodies were visible within the synovium. After negative results from autoimmune disease tests, the patient was recommended for surgical exploration as the patient reported a six-month increase in pain and difficulty flexing his index finger. Surgical exploration of the PIP joint and adjacent proximal and middle phalanx revealed several small, evenly sized, firm, smooth, creamy-white nodules. Post-op, the patient was given a splint cast for a span of seven days, followed by physical therapy, and the range of motion was achieved by the end of six months.

## Introduction

A very rare condition that affects the small joints is called synovial chondromatosis. Osseocartilaginous nodules, which result from the synovial sheath's nodular proliferation, are what define it. Following their detachment, the fragments of these nodules within the joint may grow abnormally and may calcify or ossify [1]. It is most commonly observed in the body's large joints, such as the knee, hip, elbow, and shoulder joints [2]. It is extremely uncommon in the body's small joints, such as the hand's interphalangeal and metacarpal joints and thumb [3].

It is most commonly present in the middle ages and generally affects males more than females [4]. The patient may be asymptomatic but may present with symptoms such as pain, swelling, stiffness, and decreased range of motion in the affected joints. As the symptoms are not specific and the diagnosis might be missed, careful observation and examination should be made to reach the actual diagnosis.

The illness typically resolves on its own. Conservative management for the impairment and dysfunction of the aforementioned joint includes the use of nonsteroidal anti-inflammatory drugs (NSAIDs), activity modification, and cryotherapy. Interventional treatment includes surgical management, which is the gold standard for treating synovial chondromatosis and entails removing loose bodies with or without synovectomy [5].

The purpose of this case report is to provide a comprehensive overview of the symptoms, diagnostic process, and treatment plan for a patient diagnosed with synovial chondromatosis. By detailing the clinical presentation, investigative procedures, and therapeutic interventions, we tried to enhance our understanding of this rare condition and its management.

## Case presentation

A 60-year-old woman presented to the outpatient department complaining of a six-month history of pain and swelling of the right index finger and painful limitation of the finger flexion secondary to this condition. She denied a prior history of physical trauma, ruling out the possibility of post-traumatic arthritis. We tested her for a number of autoimmune conditions that mimicked the symptoms she had presented with in order to rule out autoimmune pathology. Rheumatoid factor, nuclear antibody, neutrophil cytoplasmic antibody, citrullinated protein, and cardiolipin were among the tests, all of which turned out negative. Levels of erythrocyte sedimentation rate and C-reactive protein were also within normal limits. The differential diagnoses include rheumatoid arthritis, chronic infection (including tuberculosis), osteochondritis dissecans, osteoarthritis, neuropathic arthritis, and gout. 

**Figure 1:**
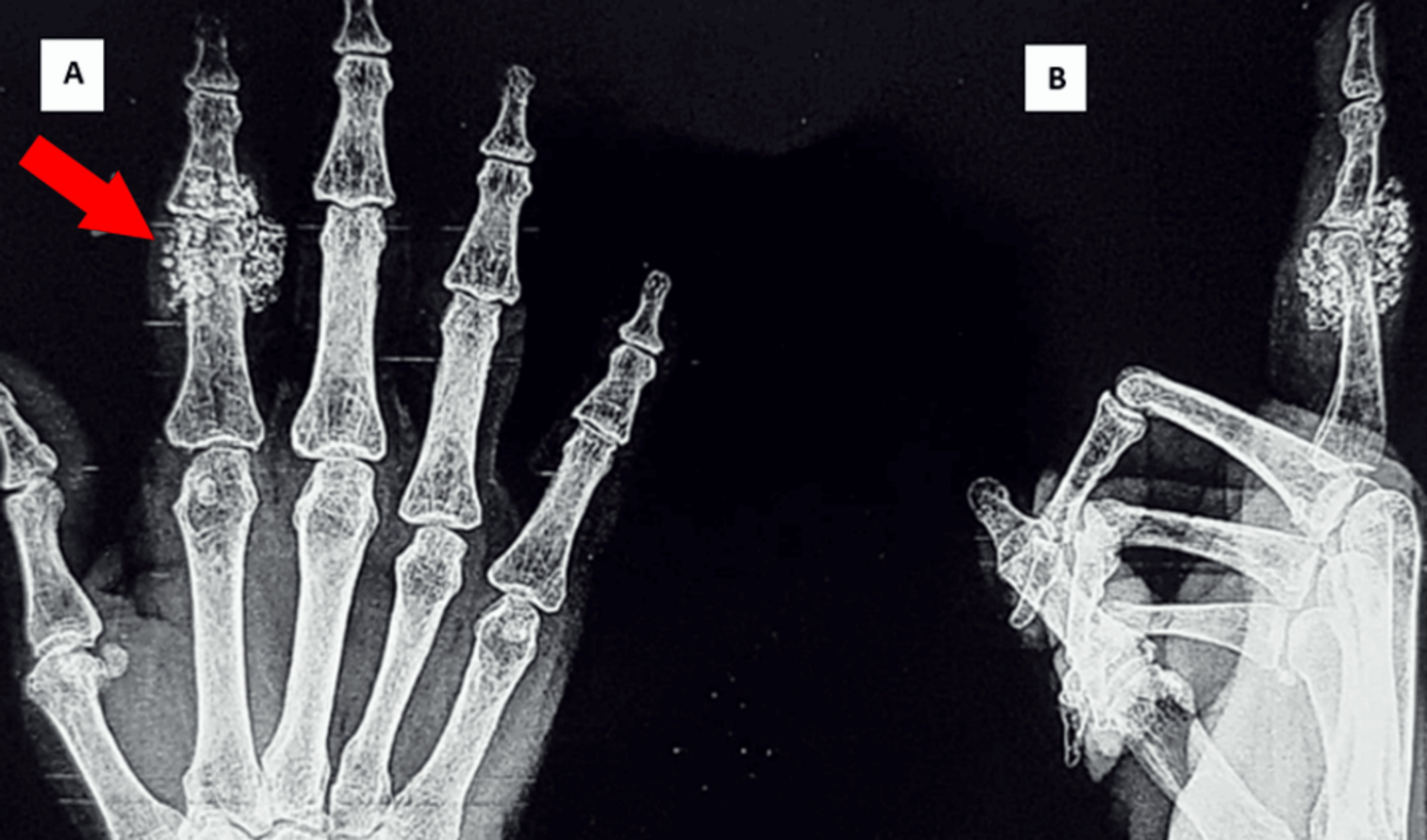
X-ray AP/laterial views of the right hand 1A: X-ray AP view of the right hand with a red arrow showing calcifications around the proximal interphalangeal joint of the index finger. 1B: X-ray lateral view of the right index finger showing calcifications.

X-rays showed multiple small, calcified nodular bodies at the level of the PIP joint, adjacent proximal, and middle phalanx of the index finger (Figure 1). Magnetic resonance imaging of the right index finger was done.

**Figure 2:**
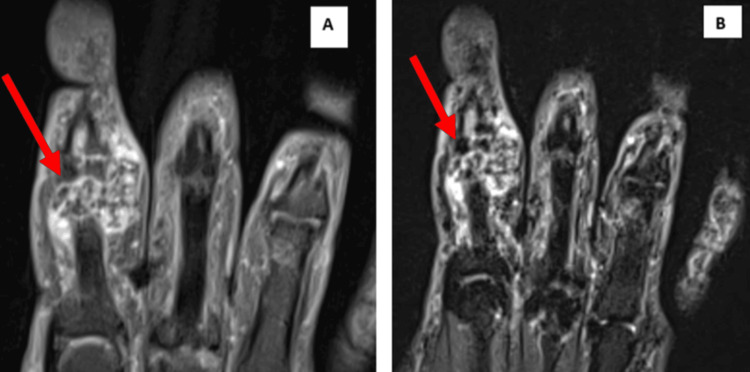
Proton density fat-saturated MRI sequence (PDFS) and short tau inversion recovery (STIR) images of the lesion near the proximal interphalangeal joint of the index finger 2A: Proton density fat-saturated MRI sequence (PDFS) and red arrows show heterogeneously hyperintense areas. 2B: Short tau inversion recovery (STIR) and red arrows show the heterogeneously hyperintense area with hypointense areas within.

The MRI images show multiple foci of blooming within due to calcifications (Figure 2).

**Figure 3:**
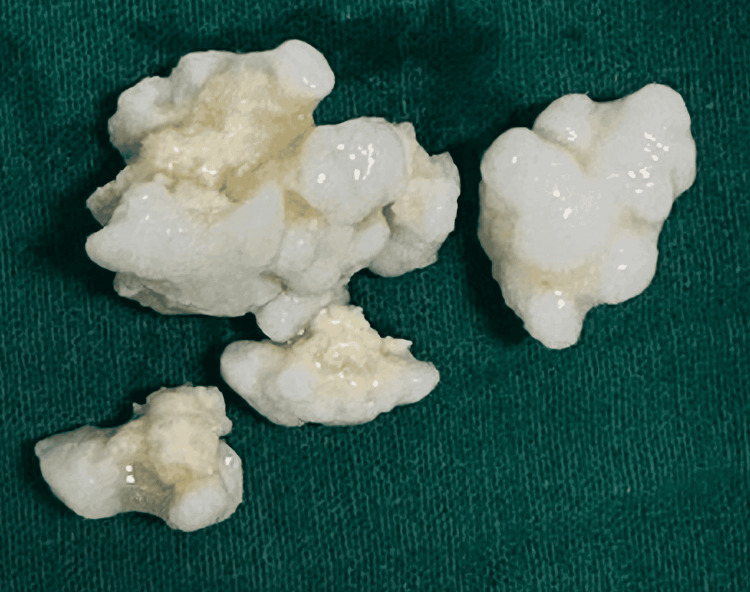
Calcified lesions from the PIP joint after debridement

After that, the patient was recommended for surgical exploration. A straight dorsal incision starts at the MCP joint and ends at the distal part of the pip joint. Care is taken not to injure the venous plexus and dorsal sensory branches of radial and ulnar nerves. The incision was placed between the lateral band and the central slip of the extensor tendon and retracted. It revealed a large encapsulated soft tissue tumour, which revealed several small, even-sized, firm, smooth, creamy-white nodules, followed by synovectomy. These nodules were sent for histopathological analysis (Figure 3).

**Figure 4:**
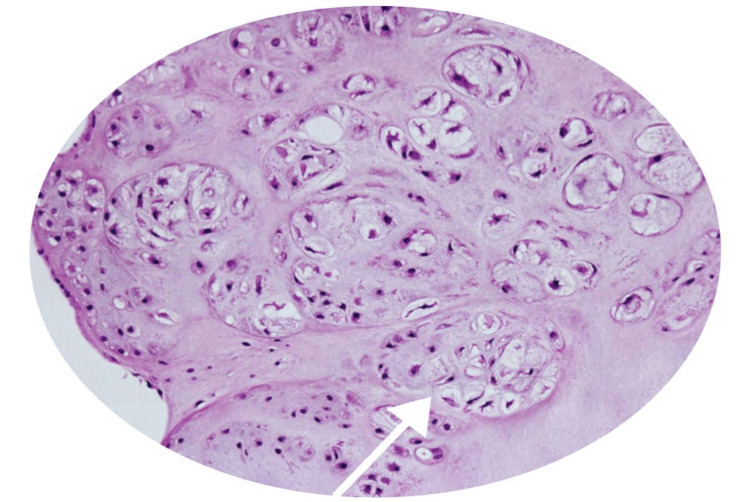
Histological examination of the excised tissue showing that the resected nodules comprise areas of the mature hyaline cartilage surrounded by a thin synovial membrane White arrow showing a cluster of chondrocytes in H and E staining (100x).

Vertical capsulotomy of the PIP joint followed by synovectomy was done. After being removed, they were sent for histopathological analysis. Histological studies revealed well-circumcised nodules of osseocartilaginous origin (Figure 4). The cartilage was of moderate cellularity, with varying degrees of ossification, a characteristic feature of synovial chondromatosis.

**Figure 5:**
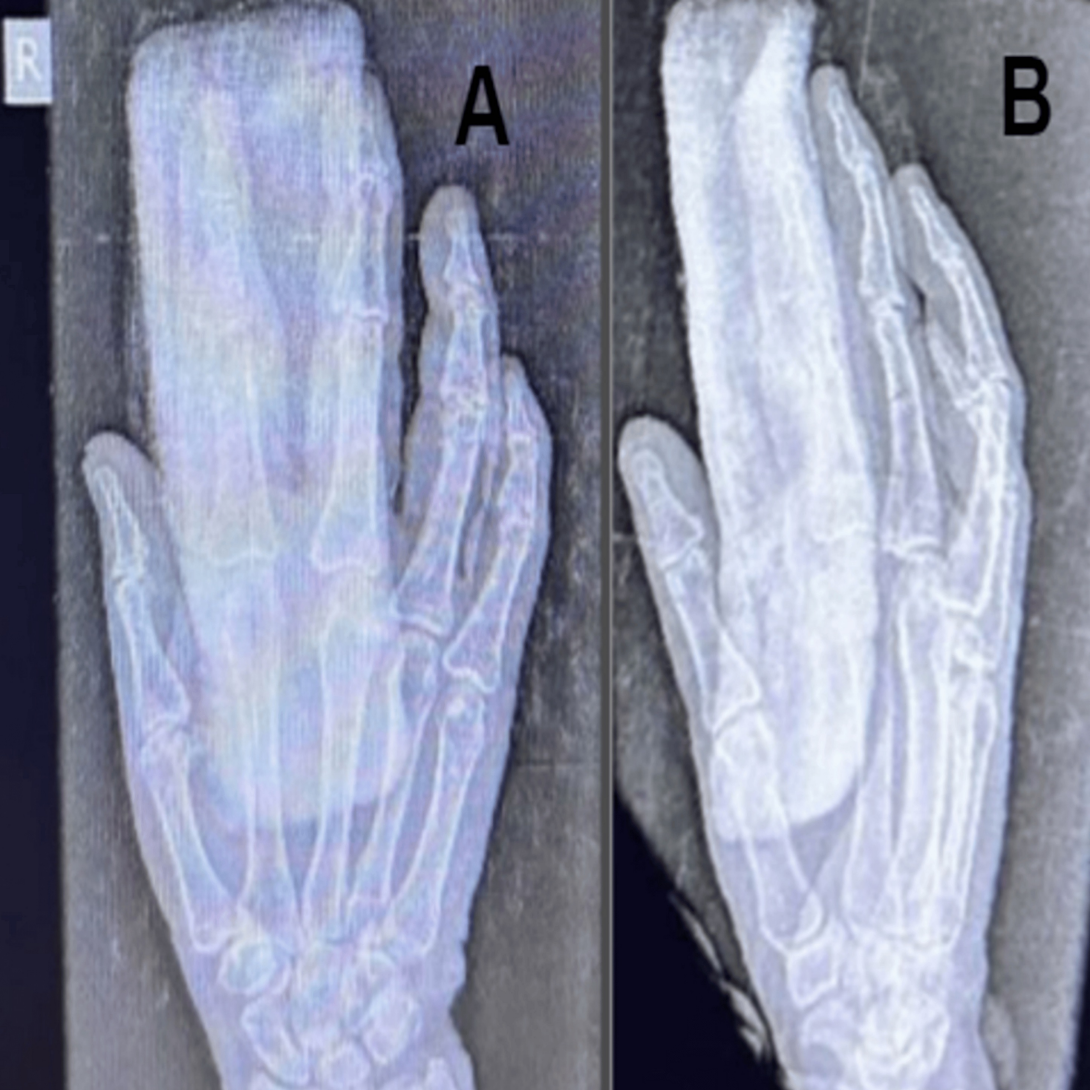
Post-op X-ray of the right hand AP and lateral view 5A: Post-op X-ray of the right hand showing the AP view; 5B: Post-op X-ray of the right hand showing the lateral view.

Post-surgery, a splint cast was applied (Figure 5). It is applied for a span of seven days and only removed for physical rehabilitation, which has been done for five days post-op to maintain the range of motion of the metacarpo-phalangeal joint.

**Figure 6:**
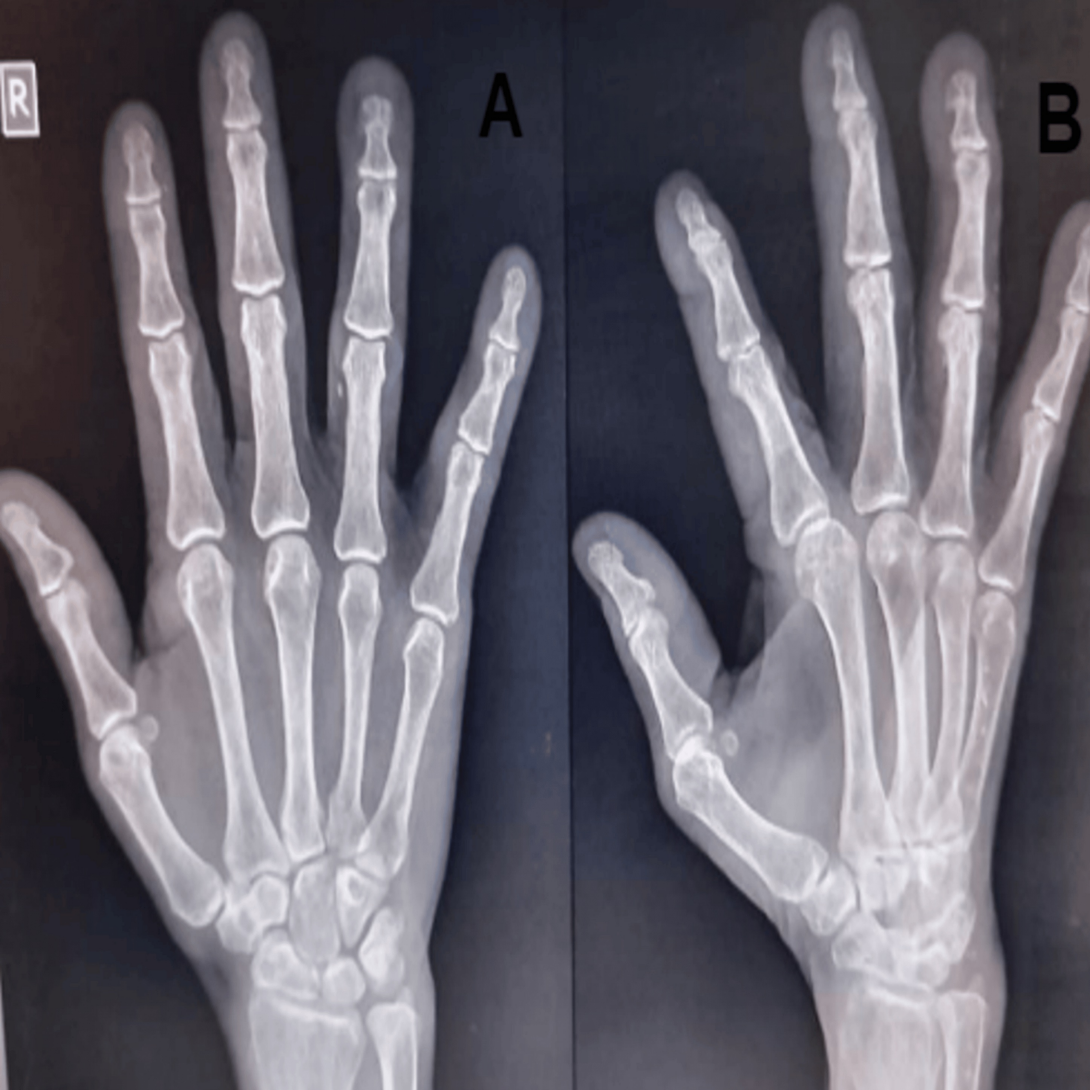
Follow-up X-ray after six months showing no recurrence

The patient was scheduled for regular follow-ups at one, three, and six months post-op. The normal range of motion was regained by the end of six months, and there was no recurrence reported (Figure 6).

## Discussion

Synovial chondromatosis is a rare, benign disorder characterized by the formation of cartilaginous nodules within the synovium. As mentioned earlier, the upper extremities are rarely affected by this illness. It is not always easy to distinguish primary synovial chondromatosis from the more prevalent secondary synovial chondromatosis, which is usually due to trauma or degenerative disease.

Its limited incidence and erratic symptoms make diagnosis difficult. Rheumatoid arthritis, psoriatic arthritis, and other autoimmune diseases are to be ruled out first. Other differential diagnoses include infection, trauma, and erosive osteoarthritis.

Although the etiopathogenesis is unknown, it is thought to result from undifferentiated mesenchymal stem cells multiplying in the synovium and producing cartilage nodules. The synovium is where cartilage grows, resulting in sessile nodules that frequently separate and stay as loose bodies inside the articular cavity or synovial folds. The synovial fluids feed these nodules and have the potential to calcify and even ossify over time [6-8].

The removal of the loose bodies typically relieves pain and other symptoms. Though it is debatable whether it prevents recurrence, synovectomy is also advised in the literature to lessen symptoms [3]. Because randomized studies cannot be conducted due to the low prevalence of synovial chondromatosis, it is difficult to determine the best course of treatment. For instance, there is a strong argument in the DIP joint favoring doing an immediate total synovectomy and arthrodesis [3,9].

Since recurrence is uncommon but possible, all patients should have long-term follow-ups. Furthermore, malignant transition into chondrosarcoma is considered extremely rare; 53 cases with synovial chondromatosis were found to have a relatively higher risk of developing chondrosarcoma in the literature.

Recurrence is another common complication. Recurrence rates following synovectomy and loose body removal range from 3% to 23% [10]. Hydrogen peroxide irrigation intraoperatively is another technique claimed to reduce recurrence [11-14].

When a recurrence is linked to particularly aggressive characteristics, such as fast joint growth or destruction, the surgeon needs to consider the possibility of malignant change [15]. Wide surgical margins are required in these patients since post-operative radiation therapy administration is advised.

To sum up, synovial chondromatosis is a rare benign tumor, and its localization in the hand is very uncommon. Therefore, a proper differential diagnosis is paramount to correctly diagnosing and treating the condition.

## Conclusions

Synovial chondromatosis is a very rare phenomenon seen in small joints that requires thorough clinical assessment, imaging, and investigation to rule out the exact diagnosis. Debridement and removal of loose bodies can be considered the gold standard treatment of choice, wherein the patient has reported reduced pain and increased range of motion, thereby improving quality of life. Additionally, conclusive diagnosis can be obtained from postoperative histological characterization. Thus, we can conclude that a surgeon has to ensure the complete removal of loose bodies, or a full synovectomy is indicated in recurrent cases.

## References

[1] Maurice H, Crone M, Watt I (1988). Synovial chondromatosis. J Bone Joint Surg Br.

[2] Sano K, Hashimoto T, Kimura K, Ozeki S (2014). Articular synovial chondromatosis of the finger. J Plast Surg Hand Surg.

[3] Harvey FJ, Negrine J (1990). Synovial chondromatosis in the distal interphalangeal joint. J Hand Surg Am.

[4] Nelson FR, Blauvelt CT (2015). Synovial chondromatosis. Orthopaedic Knowledge Update: Foot and Ankle 5.

[5] Kumar N, Mukhopadhaya J, Anand O (2016). Synovial chondromatosis of distal radioulna joint with features of lytic lesion in a 29 years male: a case report and review of literature. Sports Med Doping Stud.

[6] Isbell JA, Morris AC, Araoye I, Naranje S, Shah AB (2017). Recurrent extra- and intra-articular synovial chondromatosis of the ankle with tarsal tunnel syndrome: a rare case report. J Orthop Case Rep.

[7] Dawson-Bowling S, Hamilton P, Bismil Q, Gaddipati M, Bendall S (2008). Synovial osteochrondomatosis of the talonavicular joint: a case report. Foot Ankle Int.

[8] Arnaud JP, Girou P, Arnaud M, Pecout C (1987). [Osteochondromatosis of a distal interphalangeal articulation]. Ann Chir Main.

[9] Saxena A, St Louis M (2017). Synovial chondromatosis of the ankle: report of two cases with 23 and 126 loose bodies. J Foot Ankle Surg.

[10] Milgram JW (1977). Synovial osteochondromatosis: a histopathological study of thirty cases. J Bone Joint Surg Am.

[11] Hocking R, Negrine J (2003). Primary synovial chondromatosis of the subtalar joint affecting two brothers. Foot Ankle Int.

[12] Shpitzer T, Ganel A, Engelberg S (1990). Surgery for synovial chondromatosis. 26 cases followed up for 6 years. Acta Orthop Scand.

[13] Jonckheere J, Shahabpour M, Willekens I, Pouliart N, Dezillie M, Vanhoenacker F, De Mey J (2014). Rapid malignant transformation of primary synovial chondromatosis into chondrosarcoma. JBR-BTR.

[14] Gatt T, Portelli M (2021). Recurrence of primary synovial chondromatosis (Reichel’s syndrome) in the ankle joint following surgical excision. Case Rep Orthop.

[15] Brucher N, Faruch-Bilfeld M, Molinier F, Brouchet-Gomez A, Lapegue F, Sans N (2014). Primary synovial osteochondromatosis of the first interphalangeal joint of the foot: a case report. Diagn Interv Imaging.

